# Improving undergraduate education of occupational health and occupational medicine appling massive open online courses & problem-based learning

**DOI:** 10.1186/s12909-024-05179-7

**Published:** 2024-02-23

**Authors:** Rui Ding, Han Cheng

**Affiliations:** 1 Department of Occupational and Environmental Health, School of Public Health, Anhui Medical University, 81 Meishan Road, 230032 Hefei, Anhui China; 2 First School of Clinical Medicine, Anhui Medical University, 81 Meishan Road, 230032 Hefei, Anhui China

**Keywords:** Massive Open Online courses, Problem-based learning, Occupational Health and Occupational Medicine, Learning efficacy

## Abstract

**Background:**

The learning of *Occupational Health and Occupational Medicine* in undergraduate college students in China has been hindered by various factors. This study aimed to explore the effects of the application of Massive Open Online Courses (MOOC) & Problem-based learning (PBL) in the learning of *Occupational Health and Occupational Medicine* in undergraduate college students in China.

**Methods:**

Students enrolled in 2017 and 2018 were categorized in the control group and study group, and received PBL learning and MOOC + PBL learning, respectively. The effects of learning were assessed by scores of final exam, satisfaction degree of students, and feedbacks.

**Results:**

The mean score of the final exam was not significantly different between the two groups. However, the further comparison by levels of scores showed that the percentages of good and excellent were both significantly higher in the study group than control group. The overall satisfaction degree was significantly higher in the study group than control group. In addition, the scores of the 3 dimensions of satisfaction degree, i.e. learning preparation, learning process, and learning effect, were all significantly higher in the study group than control group. The feedbacks of the students showed that they thought MOOC + PBL learning could better improve the learning efficacy, despite a substantial proportion of students reported that MOOC + PBL learning more time-consuming.

**Conclusions:**

The findings showed that the combination of MOOC and PBL in the learning of *Occupational Health and Occupational Medicine* is an effective method capable of improving the learning efficacy in college students of *Prophylactic Medicine*. Further efforts are needed to optimize the MOOC platform to provide a friendlier interface.

## Background

*Occupational Health and Occupational Medicine* is an essential course for the education of occupational health and safety in China, which mainly focuses on the adverse effects of occupational hazards on occupational population (including the recognition, assessment, prediction, and control of adverse occupational conditions), improvement of occupational health, and diagnosis and treatment of occupational diseases [1]. However, the conventional education mode in China, i.e. students listen to the teachers, learn knowledge by observation, understanding, and reciting, has broken the inherent link between theories and practices, unintentionally. As a result, a lot of students can obtain very high scores in the end-of-course examination, but may not be capable of coping with practical problems [2]. Especially in the recent decade where numerous new manufacturing techniques have been invented, the challenges posed on the future professionals on occupational health and safety are increasing.

Although *Occupational Health and Occupational Medicine* has been included as an independent curriculum for undergraduate students of *Prophylactic Medicine*, there are several disadvantages in the teaching of this course as follows: (1) the course mainly aims to improve the health of the occupational population; however, it is increasingly difficult to give the students a chance to have a glimpse of the related occupational production [3]; (2) the learning of *Occupational Health and Occupational Medicine* requires the master of relevant knowledge of physiology, pharmacology, toxicology, epidemiology, statistics, etc., which could be of bit difficult for some students since such subjects were taught 1–3 years earlier (in their second to fourth year in the university); (3) the teaching of *Occupational Health and Occupational Medicine* mainly utilizes didactic lecture based on the textbook, which is effective for knowledge transfer, but holding student’s attention throughout the lecture is challenging [4]; and (4) the capability of students in putting knowledge into practice to cope with practical problems of occupational health or occupational medicine is not fully improved. As a consequence, students generally do not realize that they are responsible for their own learning process and self-development, thus lack the interest and may not pay enough attention on this course [5].

Problem-based learning (PBL) is a student-centered teaching mode that allows students to actively participate and interact with classmates in small groups to define the goals of learning, engage in self-study, put new knowledge into practices, and eventually improve the skills and capacities of coping with specific scenarios [6, 7]. Various previous studies have demonstrated that PBL mode can help the students to improve various abilities, such as teamwork, self-learning, communication, self-confidence, self-assessment, critical thinking, and interpersonal skills [8, 9].

However, several disadvantages have also been reported in studies adopting the PBL mode in China. For instance, PBL requires the analysis and discussion of the cases by students in small groups, while the relatively limited knowledge preparation could hinder this process [10]. Thus in most classes, especially in the teaching of *Occupational Health and Occupational Medicine*, the cases and problems only serve as a key to trigger the interest of students, but the intended goals, i.e. the improvement of certain capabilities through the processes, are not met. Furthermore, due to the heterogeneity of students regarding the relevant knowledge, the discussion in the PBL is difficult to proceed, which often ends up in the explanation by only the teacher [11]. Therefore, it is important to improve the knowledge preparation before the class, which can help maximize the efficacy of PBL.

Massive open online courses (MOOC) has been rapidly developed in China in the past several years, especially during the pandemic of coronavirus disease 2019 (COVID-19) [12]. People, not only students in colleges, can access MOOC anytime through internet, free of charge, to learn the course they are interested in [13–16]. Therefore, MOOC has become a popular educational mode in China [17, 18]. Comparing with traditional face-to-face education, MOOC allows all learners to access by internet connection without enrollment restrictions. The platform generally allows learners to watch the videos, participate in discussion, and provide feedbacks. However, the low completion rate has been stressed by various studies utilizing MOOC. A previous study reported that the completion rate of MOOC registrants was lower than 10% [19]. Such low completion rate implies the waste of learner’s time and providers’ efforts. It is urgent to take effective measurements to improve the completion rate of learners, and thus maximize the efficacy of MOOC learning.

In the past decade, various teaching modes have been applied in the learning of *Occupational Health and Occupational Medicine* in China, such as PBL, team-based learning, flipped classroom, MOOC, etc., which all improved the learning effects in various extents. Previous studies have further demonstrated the advantages of blended learning in medical education comparing to traditional class teaching methods [20]. However, the effect of combining MOOC and PBL in the learning of *Occupational Health and Occupational Medicine* in medical students has not been investigated to date. As MOOC can help students to improve the knowledge preparation before classes, and thus promote the discussion in PBL, while task-based MOOC learning could also help increase the completion rate of MOOC learning, we hypothesized that the learning mode of combining MOOC with PBL (MOOC + PBL) could improve the effects of learning of *Occupational Health and Occupational Medicine* in college students of *Prophylactic Medicine*. Therefore, this study developed the mode of combining MOOC with PBL (MOOC + PBL) based on the MOOC course, which was applied in the learning of *Occupational Health and Occupational Medicine*, aiming to evaluate the effect of MOOC + PBL mode in the learning of *Occupational Health and Occupational Medicine* in college students of *Prophylactic Medicine*.

## Subjects and methods

### Subjects

Students of *Prophylactic Medicine* enrolled in 2017 and 2018 were included in Anhui Medical University. The investigator, who was also the teacher of the students, explained the study project to the students at the beginning of the first class, and then invited the students to participate in this study. The inclusion criteria were as follows: (1) were undergraduate students of *Prophylactic Medicine* in Anhui Medical University; (2) had already completed the learning of relevant subjects, such as physiology, pharmacology, toxicology, epidemiology, statistics, etc.; (3) had a smart phone or computer and could access the internet; and (4) volunteered to participate in this study and signed informed consent. The exclusion criteria were as follows: (1) missed one third or more of the classes; and (2) did not participate in the final exam or complete the questionnaire. The study protocol was approved by Anhui Medical University’s ethical committee (20,210,052). Informed consent was obtained from all subjects.

According to the curriculum of the university, students of *Prophylactic Medicine* participate in the learning of *Occupational Health and Occupational Medicine* in the fourth year. As the MOOC designed by our team was not available online until August 2021, students enrolled in 2017 received conventional PBL, and were considered as the control group; while students enrolled in 2018 received MOOC + PBL, and were considered as the study group.

### Study design

**Fig. 1:**
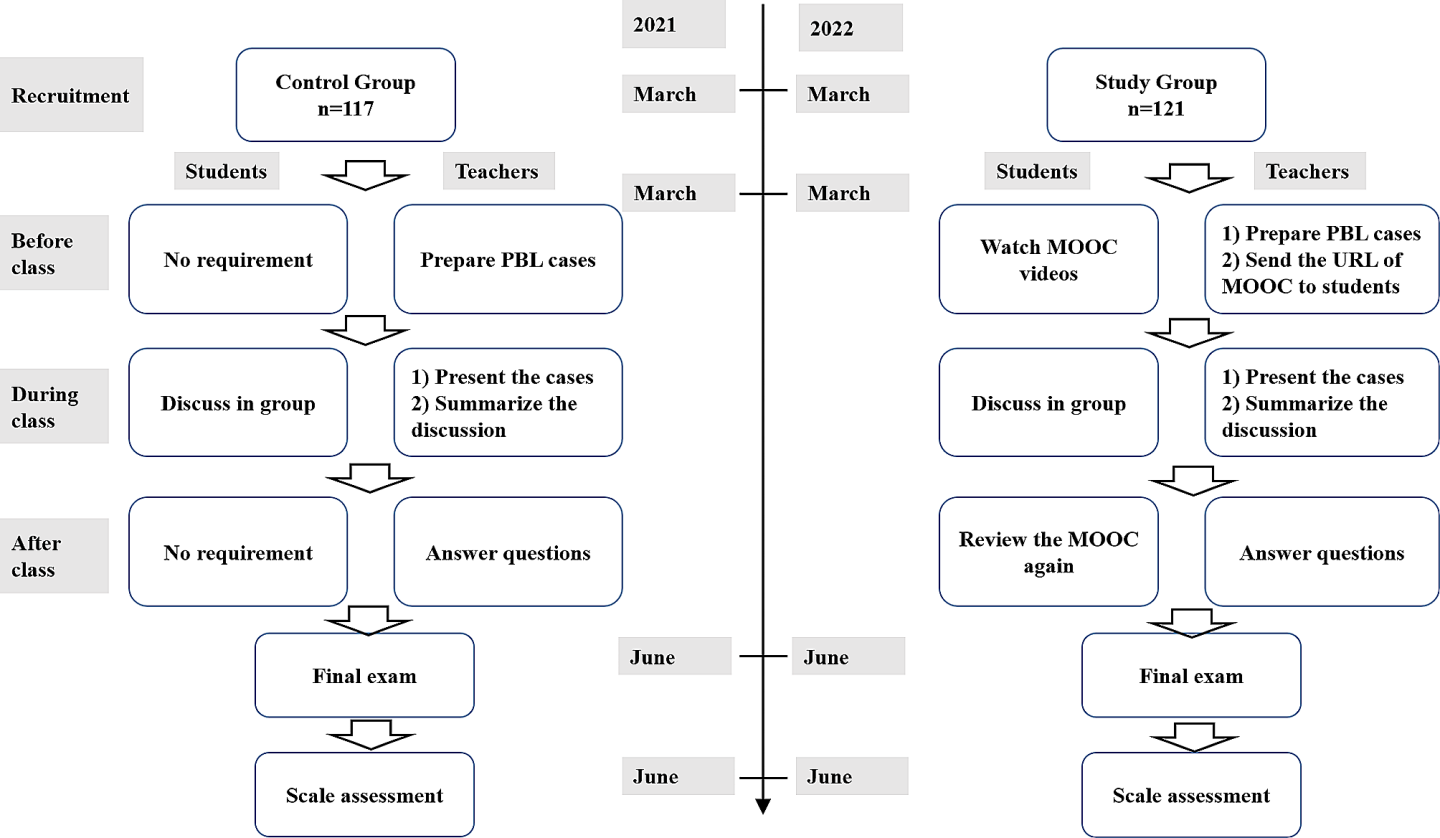
Procedures of the learning mode in the two groups

This quasi-experiment consisted of two independent parts, each of which lasted for over a period of five months, corresponded to one semester. The study in the control group was conducted in the first part (from March to July, 2021), which was based on the conventional PBL. The study in the study group was conducted in the second part (from March to July, 2022), which was based on the MOOC + PBL mode. No parallel control group was used in this study to avoid contamination, as the students could use the MOOC for learning spontaneously. Students in both groups completed the same scale for assessment after the final exam. Figure 1 shows the procedures of the learning mode in control and study groups.

### Learning mode

General data, including gender, age, source (rural area / urban area), and year of enrollment, of all participants were recorded at the beginning of the first class. For students in the control group, the conventional PBL was applied for the learning. In brief, the teachers prepare the PBL cases and questions before the class, which were then presented in the class. After the teachers explained the corresponding knowledge, the students were asked to discuss in groups to solve the questions. Finally, the teachers used the pre-prepared PowerPoint (PPT) to explain the cases and questions, and summarized the discussion findings of the students.

For students in the study group, the MOOC + PBL mode was applied. The MOOC of *Occupational Health and Occupational Medicine* has already been released by us on the platform of “Smart Education of Anhui (eHuixue)” (https://ehuixue.cn/index/detail/index?cid=37868) in August 2021. The MOOC contained 47 videos, covering all the contents of the textbook of *Occupational Health and Occupational Medicine*, with each video lasted for 10–15 min. The total time of the MOOC videos was 515 min. The teachers informed the students the contents to be learned in the forthcoming class, and sent the URL of corresponding MOOC to the students through a WeChat group one week before the class. The students then used their own smart phones or computers to access the URL and watched the videos in the MOOC by themselves, and learnt the corresponding knowledge through the internet. In the class, the teachers presented the PBL cases and questions, and guided the students to discuss in groups. Afterwards, the teachers used the pre-prepared PPT to explain the cases and questions, and summarize the discussion findings of the students. Finally, the students review the MOOC again by themselves to strengthen the understandings.

### Assessment

The effects of the learning were assessed by the satisfaction degree of the students to the teaching, as well as the analysis of scores of students in the final exam. To assess the satisfaction degree, a Likert 4-level scale was designed, which consisted 3 dimensions, i.e. learning preparation, learning process, and learning effects, with 2, 3, and 3 items included, respectively (Table 1). The answers to each item included highly satisfactory (4 points), satisfactory (3 points), common (2 points), and dissatisfactory (1 point). The scale was dispatched to the students after the final exam of the course, and the students were asked to complete the scale independently and anonymously. The score of each dimension was calculated by adding up the scores of items, and then dividing which by the number of items in the dimension. The total score of the scale was calculated by adding the scores of dimensions, and then dividing which by 3 (number of dimensions). There was also an open question (What are your suggestions/what do you think about the learning mode?) in the scale to allow students describe the limitations of the learning mode, or provide suggestions to help improve the learning mode. The scale was pilot tested in a small group of year 5 medical students of *Clinical Medicine* (*n* = 35), and the validity and reliability of the scale were assessed. The Cronbach’s α of the scale was 0.8472, indicating that the internal consistency of the scale was high. No further modifications were made to the scale.

**Table 1:** Satisfaction degree scale for the learning mode

Dimension	Item	Highly satisfactory	Satisfactory	Common	Dissatisfactory
**Learning preparation**	1) The platform for the learning meet the requirements of students.				
	2) The objects of learning are clear.				
**Learning process**	1) The students and teachers have active interactions in the learning process.				
	2) The discussions during the learning process can proceed smoothly and successfully.				
	3) The explanations to the key points and difficult points are sufficient.				
**Learning effect**	1) This learning mode can effectively improve my interest in learning.				
	2) This learning mode can improve my skills in solving problems of occupational health and occupational medicine.				
	3) This learning mode can effective improve the learning efficacy and self-study capability.				

### Statistical analysis

The Statistical Package for Social Sciences (SPSS) Version 22.0 was used for the statistical analysis in this study. Kolmogorov–Smirnov test was used for the normality test of the quantitative data, which were then described by mean and standard deviation (SD), and compared by independent t-test. Qualitative data were collected by the open question in the assessment scale. After the scale was collected, the researcher repeatedly studied the texts to discover the meaningful descriptions, in which the fixed patterns were discovered and then the coding category was developed. The codes were reviewed repeatedly, and the similar views were categorized into the same category. The interview data were then considered qualitative data for analysis, which were described by frequency and percentage (%), and compared by chi-square (χ^2^) test. *P* < 0.05 was considered statistically significant.

## Results

### General characteristics

In this study, all students of *Prophylactic Medicine* in the Anhui Medical University that enrolled in 2017 (*n* = 117) and 2018 (*n* = 121) were included, for whom the course of *Occupational Health and Occupational Medicine* was in March to June 2021 and March to June 2022, respectively. All the students completed the curriculums of the first 3.5 years before starting the learning of *Occupational Health and Occupational Medicine*, and the time of learning OHOM was both 99 class hours (including 54 class hours of theoretical lessons and 45 class hours of experimental lessons). The students in the control group included 69 boys (58.97%) and 48 girls (41.03%), and 47% of them were from rural areas. The students in the study group included 76 boys (62.81%) and 45 girls (37.19%), and 52% of them were from rural areas. The differences between the two groups were not statistically significant (*P* > 0.05).

### Test scores

After the curriculum finished, the scores of the students in the final exam were compared. The test papers and suggested answers were prepared by the same team of teachers, which were discussed and evaluated to ensure that the distribution of knowledge points, types of questions, and degree of difficulty were comparable between the two groups. The final exams in both groups were in early June in 2021 and 2022, respectively, and lasted for 120 min. The test papers were reviewed and scored by the teaching team, according to the pre-prepared suggested answers. The analysis showed that the scores in the control group (47–96 points) and study group (61–98 points) were not significantly different (78.64 ± 9.84 vs. 80.74 ± 9.16; *P* = 0.091). However, when further dividing the scores into 4 levels (< 60, fail; ≥60 but < 75, fair; ≥75 but < 85, good; and ≥ 85 but ≤ 100, excellent), the analysis showed that the percentages of good and excellent were both significantly higher in the study group than control group (Table 2).

**Table 2:** Test scores of the students in the two groups

	Control group (*n* = 117)	Study group (*n* = 121)
Mean, SD	78.64 ± 9.84	80.74 ± 9.16
< 60	1 (0.86%)	0 (0%)
60-	38 (32.48)	18 (16.70%)^*^
75-	40 (34.18%)	49 (45.39%)^*^
85–100	38 (32.48)	41 (38.00%)^*^

^*^P < 0.05, comparing with the control group

### Satisfaction degree

In the satisfaction degree evaluation, 117 and 121 questionnaires were dispatched after the final exam for the control group and study group, respectively, all of which were recovered and considered valid (Table 3). The score for the dimension “learning preparation” was 3.64 ± 0.025 in the study group, and 3.31 ± 0.036 in the control group, and the difference was statistically significant (*P* < 0.001). The score for the dimension “learning process” was also significantly higher in the study group than control group (3.22 ± 0.109 vs. 2.11 ± 0.026; *P* < 0.001). The score of the dimension “learning effect” was 3.28 ± 0.029 in the study group, which was significantly higher than the 2.63 ± 0.024 in the control group (*P* < 0.001). The total score was 3.35 ± 0.042 in the study group, which was also significantly higher than in the control group (2.61 ± 0.016; *P* < 0.001) (Table 3).

**Table 3:** The self-assessment satisfaction scores of students to the two learning modes (mean ± SD)

	Control group (*n* = 117)	Study group (*n* = 121)
Learning preparation	3.31 ± 0.036	3.64 ± 0.025^**^
Learning process	2.11 ± 0.026	3.22 ± 0.109^**^
Learning effect	2.63 ± 0.024	3.28 ± 0.029^**^
Overall satisfaction	2.61 ± 0.016	3.35 ± 0.042^**^

^**^P < 0.001, comparing with the control group

### Students’ feedbacks

The feedbacks of students were collected and analyzed. Most students (74.36%, *n* = 87) in the control group reported that “*It is difficult to do the discussion due to the insufficient knowledge preparation before the class*”, while only 22.31% (*n* = 27) in the study group reported difficulties in the discussion processes due to insufficient knowledge preparation. Almost all students (87.18%, *n* = 102) in the control group reported “*I think the cases very interesting, but I am not capable of analyzing the cases from the aspect of Occupational Health and Occupational Medicine in the class*”. In contrast, 71.07% (*n* = 86) of the students in the study group reported “*I think the MOOC-guided self-learning before the class highly efficient, it makes me capable of analyzing the cases professionally*”. However, 56.20% (*n* = 68) also complained that “*The MOOC-guided self-learning is time consuming, I have to spend a lot of additional time on it before the class*”, while only 36.75% (*n* = 43) reported spending substantial time on self-learning before the class. In addition, 75 (61.98%) students in the study group reported “*The MOOC platform allows me to do the learning whenever I am free*”; however, 94 (77.69%) of the students in the study group also reported “*Every time log in to the MOOC platform requires the input of account, password, and telephone number drives me crazy*”.

## Discussion

Through the analyses from three aspects, i.e. test scores, satisfaction degree, and feedback, this study investigated the effect of MOOC + PBL mode in the learning of *Occupational Health and Occupational Medicine* in college students of *Prophylactic Medicine*. The findings showed that comparing with PBL learning mode, the MOOC + PBL mode effectively improved the learning effects.

Despite the various advantages, PBL still involves several disadvantages that limit the application in college students [21]. For instance, the presentation of cases as well as corresponding questions in the class for analysis and discussion requires sufficient knowledge reserve [22], especially in the course of *Occupational Health and Occupational Medicine*. In our practices of utilizing PBL in the teaching of *Occupational Health and Occupational Medicine*, the class was a bit “too silent” as the students did not know where to start and how to discuss in depth, and the teachers had to answer and explain almost all the questions by themselves. Most students reported “*feeling awkward and dare not to look into the eyes of teachers*” as they were afraid of being asked to provide their opinions, despite that they acknowledged that the cases were interesting and the teaching based on the cases and problems more helpful than the learning processes not using PBL. However, the eventual goal of improving the learning efficacy of students could not be achieved in this impaired “PBL” mode.

The findings of this study demonstrated that the disadvantages of PBL learning could be, at least partly, overcome by MOOC + PBL learning. In the practices of MOOC + PBL, the students were instructed to watch the videos about 1 week before the class, which allowed students to go over the relevant knowledge and thus improve the knowledge preparation before the class. Such self-learning of the students made it possible to analyze and discuss the cases and problems presented in the class. The successful discussion on cases in turn further improved the confidence of students and reinforced the interests in self-learning. Comparing with the control group that utilized PBL mode in this study, students in the study group that utilized MOOC + PBL mode actively participated in the discussion, provided relatively professional opinions on the case and questions, and had active interactions with peer-students and teachers. According to the feedbacks of students, they had high sense of accomplishment and satisfaction. Comparing with the conventional teaching mode that utilized no MOOC or PBL, MOOC + PBL mode effectively improved the initiative of learning in the students, but also helped the students to put the knowledge into analyzing the practical problems.

It has been well acknowledged that the completion rate of MOOC learning is very low, leading to the waste of students’ time, providers’ efforts, and platform resources [19]. The lack of “learning environment”, i.e. direct communications with teachers and peer-learners, encouraging comments, and constructive suggestions, makes learners feeling lonely; the lack of further explanations of knowledge and the chance of putting the knowledge into practices also contributes to the high rate of drop out [23].

During the adoption of MOOC + PBL in this study, the students were clearly required to watch the videos 1 week before the corresponding class. Different from spontaneous MOOC-based learning, using MOOC for self-learning beforehand was imposed as a task, and the teachers kept in touch with students through the MOOC platform as well as other platforms (such as QQ and WeChat) 2–3 times per week, which guaranteed the completion rate of MOOC learning. The intention of participating in the analysis and discussion on the cases and problems in the class also urged the students to actively do the MOOC-based self-learning. It is difficult to explain the production technology processes to the students, thus linking the theoretical knowledge to the practices in the real world is very hard [24]. In the MOOC + PBL learning mode, videos and pictures, along with the explanations of teachers and engineers from factories, can be used to illustrate the processes of production. Then the PBL-based analysis and discussion in the class could help the students link the knowledge to production processes, and thus strengthen the understandings of the knowledge, eventually enhance the capability of analyzing and coping with practical issues in occupational production. In this study, the learning effect of students was significantly higher in the study group (using MOOC + PBL learning) than control group (using PBL learning), demonstrating the advantage of MOOC + PBL mode than PBL mode. A substantial proportion of students in the study group also reported “*high self-confidence*” and “*sense of achievement*” after the MOOC + PBL learning.

Despite the advantages of MOOC + PBL learning that illustrated in this study, several disadvantages were also shown. For instance, the MOOC platform was not very friendly, and 77.69% of the students in the MOOC + PBL group reported that the tedious log in processes every time using MOOC for learning “drove them crazy”. After the final exam of the course, we also asked a group of students about the suggestions. Some students reported that “*Every time thinking of using the MOOC for the learning, the tedious log in process prevented me. If not imposed as a task, I may have already given up long ago*”. This opinion was immediately agreed by other students. In addition, although MOOC allows learners to learn anytime, 56.2% of the students in the study group felt the learning “time consuming”, mainly due to the fact that the learning was imposed as a task by teachers.

## Conclusions

The combination of MOOC and PBL in the learning of Occupational Health and Occupational Medicine is an effective method that capable of overcoming the disadvantages of both MOOC and PBL learning, and eventually improve the learning efficacy and capability of putting knowledge into practice in college students of *Prophylactic Medicine*. We recommend that teachers to keep in close touch with the students during the pre-class MOOC learning when applying the MOOC-PBL mode, which is capable of not only improving the completion rate of MOOC learning, but also guaranteeing the smooth proceeding in PBL discussion. Further efforts are needed to optimize the MOOC platform to provide a friendlier interface.

## Data Availability

All data generated and analyzed during this study are included in this published article.

## Abbreviations

MOOC: Massive Open Online Courses
PBL: Problem-based learning
COVID-19: Coronavirus disease 2019
PPT: PowerPoint
SPSS: Statistical Package for Social Sciences ()
SD: Standard deviation
